# Serum dihydroceramides correlate with insulin sensitivity in humans and decrease insulin sensitivity in vitro

**DOI:** 10.1016/j.jlr.2022.100270

**Published:** 2022-08-27

**Authors:** Simona Zarini, Joseph T. Brozinick, Karin A. Zemski Berry, Amanda Garfield, Leigh Perreault, Anna Kerege, Hai Hoang Bui, Phil Sanders, Parker Siddall, Ming Shang Kuo, Bryan C. Bergman

**Affiliations:** 1 Division of Endocrinology, Metabolism, and Diabetes, University of Colorado Anschutz Medical Campus, Aurora, Colorado, USA; 2 Division of Eli Lilly and Co., Lilly Research Laboratories, Indianapolis, Indiana, USA

**Keywords:** sphingolipids, circulating ceramides, serum, insulin resistance, lipidomics, CVD, T2D, obesity, myotube, Ath, lean endurance trained athlete, Lean, lean sedentary control, Ob, sedentary individual with obesity, Rd, rate of disappearance

## Abstract

Serum ceramides, especially C16:0 and C18:0 species, are linked to CVD risk and insulin resistance, but details of this association are not well understood. We performed this study to quantify a broad range of serum sphingolipids in individuals spanning the physiologic range of insulin sensitivity and to determine if dihydroceramides cause insulin resistance in vitro. As expected, we found that serum triglycerides were significantly greater in individuals with obesity and T2D compared with athletes and lean individuals. Serum ceramides were not significantly different within groups but, using all ceramide data relative to insulin sensitivity as a continuous variable, we observed significant inverse relationships between C18:0, C20:0, and C22:0 species and insulin sensitivity. Interestingly, we found that total serum dihydroceramides and individual species were significantly greater in individuals with obesity and T2D compared with athletes and lean individuals, with C18:0 species showing the strongest inverse relationship to insulin sensitivity. Finally, we administered a physiological mix of dihydroceramides to primary myotubes and found decreased insulin sensitivity in vitro without changing the overall intracellular sphingolipid content, suggesting a direct effect on insulin resistance. These data extend what is known regarding serum sphingolipids and insulin resistance and show the importance of serum dihydroceramides to predict and promote insulin resistance in humans.

Circulating ceramides, especially specific saturated ceramide species, and other sphingolipids are linked to CVD risk and insulin resistance (1, 2, 3, 4, 5, 6, 7, 8, 9, 10). In fact, circulating ceramide and sphingolipid contents predict development of CVD better than some common risk factors such as plasma cholesterol, LDLs, and triglycerides (6, 9, 11, 12). As a result, it was recently proposed that plasma ceramide could be the new cholesterol for assessing risk of CVD (11). Beyond the cross-sectional studies referenced above, there are several lines of evidence supporting the link between ceramides, CVD, and insulin resistance. Plasma ceramide content decreases after insulin-sensitizing gastric bypass surgery and weight loss interventions (13, 14, 15). Animal studies show that ceramides accumulate in atherosclerotic lesions, which may explain the increased risk associated with plasma content (16). However, the relationship of circulating sphingolipids to insulin resistance is not absolute, as insulin-sensitizing treatments do not always change plasma sphingolipid content (17). Combined, most data from epidemiology studies, as well as human interventions and animal models, support the concept that circulating ceramides and sphingolipids are related to insulin resistance and CVD risk.

Ceramides circulate primarily bound to lipoproteins and are secreted predominately by the liver. Circulating ceramides are mainly increased in LDL in individuals with obesity (15). Obese rodents have increased hepatic ceramide secretion, which may explain increased plasma ceramide content in individuals with obesity (15). In one mechanistic study, an LDL-ceramide mixture was infused in mice to recapitulate increased plasma ceramide content in obesity, which caused membrane ceramide accumulation, decreased insulin signaling, and a decrease in insulin sensitivity specifically in skeletal muscle, providing evidence for a direct effect of circulating ceramides on tissues (15). Similarly, LDL-ceramide administration to myotubes caused ceramide accumulation, decreased insulin sensitivity, and signaling independent of inflammation. These data indicate that plasma ceramides are not simply markers of insulin resistance but play mechanistic roles in decreasing insulin sensitivity.

Ceramides are only one member of the sphingolipid family, and other sphingolipids may also be related to insulin resistance and CVD risk. Lactosylceramides and glucosylceramides are sphingolipids that also accumulate in atherosclerotic plaques and therefore may be involved in the CVD process (18). Sphingomyelins are the most abundant sphingolipids circulating in lipoproteins and, while they are positively related to obesity and waist circumference, they are not correlated to insulin sensitivity in cross-sectional human studies (5, 19). Dihydroceramides are immediate precursors to ceramide synthesis and are negatively related to insulin sensitivity (20, 21) and insulin secretion (21), are positively related to waist circumference (22), are elevated in plasma of individuals with prediabetes and T2D compared with controls (23), and predict development of diabetes 9 years before onset (21). Despite strong evidence linking plasma dihydroceramides to decreased insulin sensitivity, mechanistic studies to determine if circulating dihydroceramides cause insulin resistance are lacking.

To address this knowledge gap, we performed the current study to assess serum sphingolipids in humans across the metabolic spectrum as well as determine if dihydroceramides induce insulin resistance in vitro.

## Materials and methods

### Subjects

Sixteen lean endurance trained athletes (Ath), 14 lean sedentary controls (Lean), 15 sedentary individuals with obesity (Ob), and 12 sedentary individuals with obesity and T2D were recruited for this study.

Subjects gave written informed consent and were excluded if they had a BMI <20 kg/m^2^ or >25 kg/m^2^ for Lean and Ath and BMI <30 or >40 kg/m^2^ for Ob and T2D, or had fasting triglycerides >150 mg/dl, liver, kidney, thyroid, or lung disease. Sedentary subjects were engaged in planned physical activity <2 h/week. Endurance athletes were master athletes training for cycling and triathlon competitions. Individuals with T2D were excluded from the study if they used insulin and/or thiazolidinediones. All other medications were permitted but washed out for 2 weeks prior to metabolic testing. These medications included metformin (*n* = 4), sitagliptin (*n* = 2), sitagliptin/metformin (*n* = 2), glimepiride (*n* = 1), glyburide (*n* = 1), glipizide (*n* = 1), and liraglutide (*n* = 2). Participants with obesity, lean controls, and athletes were not taking medications. Subjects were weight stable in the 6 months prior to the study and were asked to refrain from planned physical activity for 48 h before the metabolic study.

This study was approved by the Colorado Multiple Institutional Review Board at the University of Colorado Anschutz Medical Campus, protocol #10-0443 and abides by the Declaration of Helsinki principles.

### Preliminary testing

Subjects reported to the Clinical Translational Research Center for screening procedures following a 12-h overnight fast, where they were given a health and physical examination, followed by a fasting blood draw. Volunteers underwent a standard 75 g oral glucose tolerance test to verify glucose tolerance. Body composition was determined using dual-energy X-ray absorptiometry analysis (Lunar DPX-IQ; Lunar Corporation, Madison, WI).

### Insulin clamp study

Volunteers spend the night on the Clinical Translational Research Center to ensure compliance with the overnight fast. After a 12-h overnight fast, an antecubital vein in one arm was cannulated for infusions of glucose stable isotopes, insulin, and spiked dextrose, and a retrograde dorsal hand vein in the contralateral side was catheterized for blood sampling via the heated hand technique. A primed continuous infusion of [6,6-^2^H_2_]glucose (Cambridge Isotope Laboratories, Tewksbury, MA) was initiated at 0.04 mg/kg/min and continued throughout a 2-h basal lead in period and the insulin clamp. Blood samples for determination of baseline hormone, substrate concentrations, and serum preparation were drawn during the final 30 min of the 2-h tracer equilibration before the clamp. A hyperinsulinemic euglycemic clamp was then initiated and continued for the next 3 h using the method of DeFronzo *et al.* (24). Briefly, a primed continuous infusion of insulin was administered at 40 mU/m^2^/min for 3 h. A variable infusion of 20% dextrose was infused to maintain blood glucose ∼90 mg/dl. The dextrose infusion used to maintain euglycemia was labeled with [6,6-^2^H_2_]glucose to maintain stable enrichment of plasma glucose (25). Arterialized blood was sampled every 5 min for bedside determination of glucose concentration (Analox, Lunenberg, MA) and the dextrose infusion adjusted as necessary. Glucose rate of disappearance (Rd) during the insulin clamp was calculated using equations described by Finegood *et al.* (25) and normalized to body weight.

### Serum lipidomics analysis

LC-ESI-MS/MS analysis of serum for most sphingolipids was performed quantitatively using an AB Sciex quadrupole mass spectrometer 6500 (Sciex, Framingham, MA) equipped with an ESI probe and interfaced with the Agilent 1290 infinity LC system (Agilent, Palo Alto, CA). Sphingolipids were separated with a Poroshell 120 EC-C8 column, 2.1 × 50 mm, 2.7 μm (Agilent). Lipids from serum were extracted using 1-phase extraction (methanol-dichloromethane), after internal standard addition. Quantification was performed using the ratio of analyte to internal standards relative to sphingolipid calibration curves.

Serum triglycerides, cholesteryl esters, phospholipids, lysophospholipids, and sphingomyelins were extracted via a modified Folch extraction after internal standard addition. Analysis was conducted via flow injection ESI-MS/MS into a 5600 TripleTOF mass spectrometer (Sciex). Mass spectra were acquired in two stages. In the first stage, the TOF spectra were scanned with no fragmentation from 100 to 1,200 Da. The second stage consisted of TOF product ion scans of 611 precursor masses from 349.2 to 959.8. Lipids were identified in the second stage by precursor and product ion pairs predicted by the analyte species and lipid class. Results are reported as ratios of analyte area to internal standard area.

### Cell culture

Primary human skeletal muscle cells were seeded in 24-well plates coated with 5 μg/cm^2^ collagen I from rat tail (Corning, Inc, Corning, NY) and grown in DMEM supplemented with fetuin (0.25 mg/ml), BSA (0.5 mg/ml), gentamicin (0.025 mg/ml), amphotericin B (0.125 μg/ml), recombinant human epidermal growth factor (0.01 μg/ml), dexamethasone (0.39 μg/ml), 1× GlutaMAX, 10% fetal bovine serum, and 2% penicillin/streptomycin, at 37°C in 5% CO_2_. When cells reached about 90% confluence, they were differentiated into myotubes by switching the media to DMEM containing 2% serum. After 7 days of differentiation, cells were treated with dihydroceramide-containing liposomes, and insulin-stimulated glycogen synthesis assay was performed.

### Phospholipid/dihydroceramide liposome preparation

To mimic in vivo conditions, dihydroceramides were delivered to human primary muscle cells via liposomes, artificial spherical vesicles composed of a phospholipid bilayer. We prepared liposomes containing C18:0, C24:0, and C24:1 dihydroceramides by mixing POPC and individual dihydroceramide standards at a ratio of 8:1 (2.5 mg of POPC and 312 μg of individual dihydroceramides, in 80/20 methylene chloride/methanol). Individual dihydroceramide liposomes were mixed at the appropriate concentrations before administration to cells. Control liposomes were prepared by mixing 2.5 mg of POPC with 320 μl of 80/20 methylene chloride/methanol. Each POPC/dihydroceramide mixture was evaporated while mixing under a stream of nitrogen to form a thin film and then rehydrated with PBS. The solution was left at 4°C overnight and then sonicated twice at 37°C for 15 min. Liposome vesicles were formed using a Mini-Extruder apparatus (Avanti Polar Lipids, Alabaster, AL), by extruding the lipid solution five times through a 0.2 μm Nuclepore polycarbonate track-etched hydrophilic membrane (Cytiva Whatman, Marlborough, MA) and five more times through a 0.1 μm membrane, keeping the solutions and extruder at 37°C. The final dihydroceramide concentration of the individual liposome preparations was confirmed by LC-MS/MS.

### Cell treatment and glycogen synthesis assay

Cells were serum starved for 3.5 h in plain DMEM prior to the glycogen synthesis assay and treated with liposomes during the serum-starve period. Cells were treated with a mixture of C18:0, C24:0, or C24:1 dihydroceramide liposomes at a concentration similar to the one observed in serum from individuals with obesity or T2D (10 ng/ml for C18:0 dihydroceramide, 50 ng/ml for C24:0 dihydroceramide, and 180 ng/ml for C24:1 dihydroceramide). Liposome vesicles made without dihydroceramides (liposome control) were added at the same concentration as treated cells. Additional experiments were conducted treating the cells with the individual dihydroceramide liposomes, each at the same concentration used in the mixture.

Glycogen synthesis assay was performed according to previously published protocols (26), with some modifications. Briefly, cells were incubated for 1 h at 37°C with 2 μCi/ml D-[U-^14^C]-glucose (Cambridge Isotope Laboratories) with or without 100 nM insulin. After four washes with ice-cold Dulbecco's PBS, cells were collected in 150 μl of 1 M KOH and heated at 100°C for 10 min. About 15 μl aliquots were saved for Micro BCA protein assay (Thermo Scientific, Rockford, IL). After addition of 60 μl of a 25 mg/ml glycogen solution in water and 40 μl of saturated Na_2_SO_4_, glycogen was precipitated by adding 900 μl of ice-cold ethanol and incubating the samples overnight at −80°C. After centrifugation at 13,000 rpm for 10 min, the pellets were resuspended in 50 μl of water. After addition of 1 ml of ice-cold 70% ethanol, samples were left for at least 3 h at −80°C and centrifuged again at 13,000 rpm for 10 min. The final pellets were resuspended in 100 μl of water, transferred into scintillation vials containing 5 ml of scintillation fluid (CytoScint ES; MP Biomedicals, Solon, OH), and counted for radioactivity on a LS 6000TA scintillation counter (Beckman, Pasadena, CA).

### Ceramide LC-MS/MS analysis of primary skeletal muscle cells treated with dihydroceramide liposomes

Cells were grown and differentiated in collagen-coated 6-well plates and treated with control and mixed dihydroceramide liposomes 3.5 h. Wells were washed three times with ice-cold PBS and scraped in 500 μl of water. Cells were homogenized for 2 min at 25 Hz using a bead mill homogenizer (TissueLyser; Qiagen, Hilden, Germany). After removing 20 μl for protein measurements to normalize the results, equal volumes of homogenate were brought to 750 μl total volume with water and 900 μl of methanol were added. Internal standards (C12:0 ceramide, C12:0 dihydroceramide, C12:0 glucosylceramide, and C17:0 lactosylceramide; Avanti Polar Lipids) were added, and lipid extraction was performed by addition of 3 ml of methyl-*tert*-butyl-ether, according to Matyash *et al.* (27). Ceramides, dihydroceramides, glucosylceramides, lactosylceramides, sphingosine, and sphingomyelins were analyzed and quantitated by LC-MS/MS using a 2000 QTrap mass spectrometer (Sciex), as previously described by our laboratory (28).

### Immunoblot analysis

Proteins were separated by SDS-PAGE and transferred to a PVDF membrane. Membranes were incubated for 1 h in Intercept Blocking Solution (LI-COR, Lincoln, NE) and then incubated with the primary antibody overnight at 4°C in Intercept Antibody Diluent (LI-COR). Membranes were washed in TBS with 0.1% Tween-20 and incubated with IRDye secondary antibody (LI-COR) in Intercept Antibody Diluent for 1 h at room temperature without light exposure. The signal was detected using an Odyssey CLx Infrared Imaging System (LI-COR) and analyzed using Image Studio software (LI-COR). All antibodies were purchased from Cell Signaling Technology (Danvers, MA), except for anti-phospho-IRS1 (Tyr612) (MilliporeSigma, Burlington, MA).

### Statistical analysis

Data are presented as mean ± SEM. Differences between groups and treatments were analyzed using a one-way ANOVA (SPSS, Chicago, IL). Significant differences in individual lipid species between groups were adjusted for multiple comparisons using the Benjamini-Hochberg procedure. When significant differences were detected, groups were compared using two-tailed Student’s *t*-tests. Significant relationships between serum lipids and insulin sensitivity were determined using Pearson’s correlation coefficient and were adjusted for multiple comparisons using the Benjamini-Hochberg procedure. A *P* value of less than 0.05 was considered significant.

## Results

Demographics for the individuals in this study have already been published (29). Briefly, 16 athletes (6W/10M), 14 lean controls (6W/8M), 15 individuals with obesity (7W/8M), and 12 individuals with T2D (5W/7M) volunteered to participate in the study. A sample from one individual with obesity was not analyzed for shotgun lipidomics because of technical problems resulting in a sample size of 14 for individuals with obesity for triglyceride, cholesteryl ester, and phospholipid data. There were no differences in mean age between groups (Ath: 42.5 ± 1.3, Lean: 42.6 ± 1.9, Ob: 42.0 ± 1.5, and T2D: 45.3 ± 1.7 years, *P* = 0.46). As expected, BMI (Ath: 23.1 ± 0.5, Lean: 22.4 ± 0.7, Ob: 35.4 ± 1.1, and T2D: 34.8 ± 1.8 kg/m^2^; *P* < 0.0001) and body fat percentage (Ath: 16.8 ± 1.4, Lean: 22.8 ± 2.4, Ob: 36.7 ± 2.0, and T2D: 35.9 ± 2.6%; *P* < 0.0001) were significantly greater in Ob and T2D individuals compared with Lean and Ath. Resting systolic blood pressure was significantly greater in T2D compared with Ath and Lean (Ath: 115 ± 2.8, Lean: 113 ± 3.2, Ob: 121 ± 3.8, and T2D: 126 ± 2.5 mm Hg; *P* = 0.035), whereas there were no significant differences in diastolic blood pressure between groups (Ath: 73 ± 3.1, Lean: 71 ± 2.4, Ob: 79 ± 2.7, and T2D: 80 ± 3.5 mm Hg). Fasting glucose was significantly greater in the T2D group compared with others (Ath: 88.1 ± 2.4, Lean: 90.2 ± 1.6, Ob: 93.3 ± 2.1, and T2D: 173.7 ± 12.8 mg/dl; *P* < 0.0001), whereas insulin concentration was significantly greater in Ob and T2D compared with Ath and Lean (Ath: 6.7 ± 0.7, Lean: 8.8 ± 01.3, Ob: 18.1 ± 2.1, and T2D: 21.5 ± 2.7 kg/m^2^; *P* < 0.0001). Insulin sensitivity measured by insulin-stimulated glucose Rd was significantly different between each group (Ath: 12.4 ± 0.6, Lean: 8.8 ± 0.7, Ob: 5.1 ± 0.6, and T2D: 2.4 ± 0.4 mg/kg/min; *P* < 0.0001).

**Figure 1:**
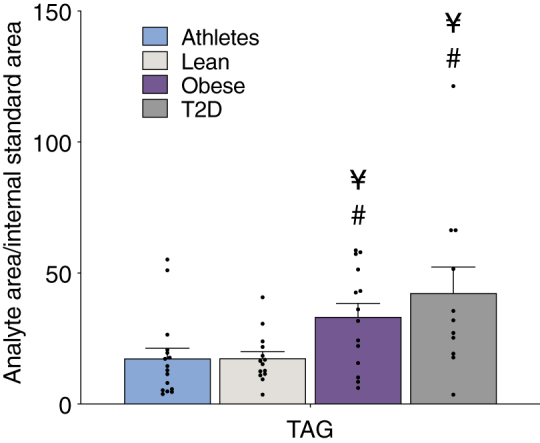
Serum triglyceride content in individuals spanning different ranges of insulin sensitivities*.* Serum triglycerides (triacylglycerol [TAG]) in endurance trained athletes, lean sedentary controls, and individuals with obesity without and with T2D. Values are means ± SEM and expressed as ratio between the analyte and the internal standard areas in 1 ml of serum. Values are means ± SEM. ¥ = significantly different than lean *P* < 0.05, # = significantly different than athletes *P* < 0.05.

Individuals with obesity and T2D had significantly greater serum triglycerides compared with Lean and Ath (Fig. 1). Triglyceride data from one individual with T2D were removed from this analysis with outlier values that were seven times the mean and 7.8 standard deviations away from the mean. There was a significant inverse relationship between insulin sensitivity measured by insulin-stimulated glucose Rd and total serum triglyceride content (*r* = −0.37; *P* = 0.006).

**Figure 2:**
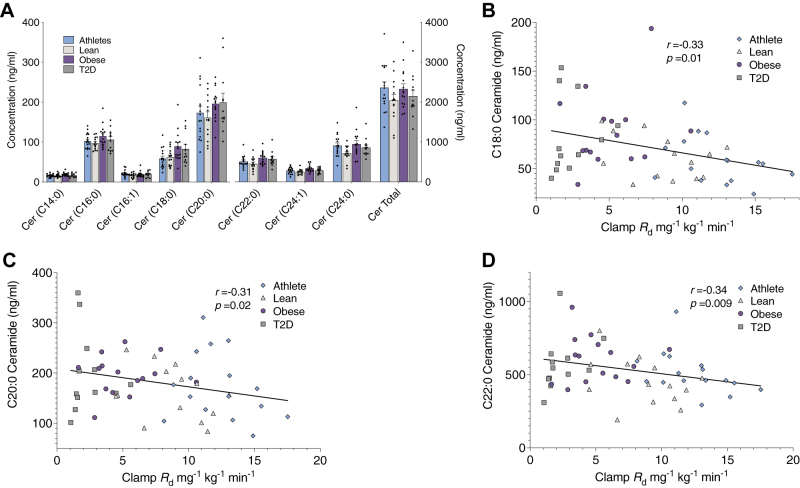
Serum ceramides and relationships to insulin sensitivity. Serum ceramide content (A) and relationship of C18:0 (B), C20:0 (C), and C22:0 (D) ceramide species to insulin sensitivity in endurance trained athletes, lean sedentary controls, and individuals with obesity without and with T2D. Values are means ± SEM.

After correcting for multiple comparisons, there were no significant differences in total serum ceramide content between groups (Fig. 2A). However, using all serum ceramide data relative to insulin sensitivity as a continuous variable, we found that C18:0, C20:0, and C22:0 ceramide species were significantly related to insulin sensitivity after correction for multiple comparisons (Fig. 2B–D). The ratio of C16:0, C18:0, and C20:0 serum ceramides to C24:0 content has been linked to CVD risk (9); therefore, we evaluated these ratios to insulin sensitivity and found that none was significantly different between groups, but C18:0/C24:0 serum ceramide ratio was significantly inversely related to insulin sensitivity (*r* = −0.33; *P* = 0.01).

There were no differences in serum cholesteryl esters, phospholipids and lysophospholipids, sphinganine, sphingosine, sulfatides, hexosyl- and lactosyl-ceramides, gangliosides and sphingomyelins between groups, and no relationships to insulin sensitivity (supplemental Figs. S1–S5).

**Figure 3:**
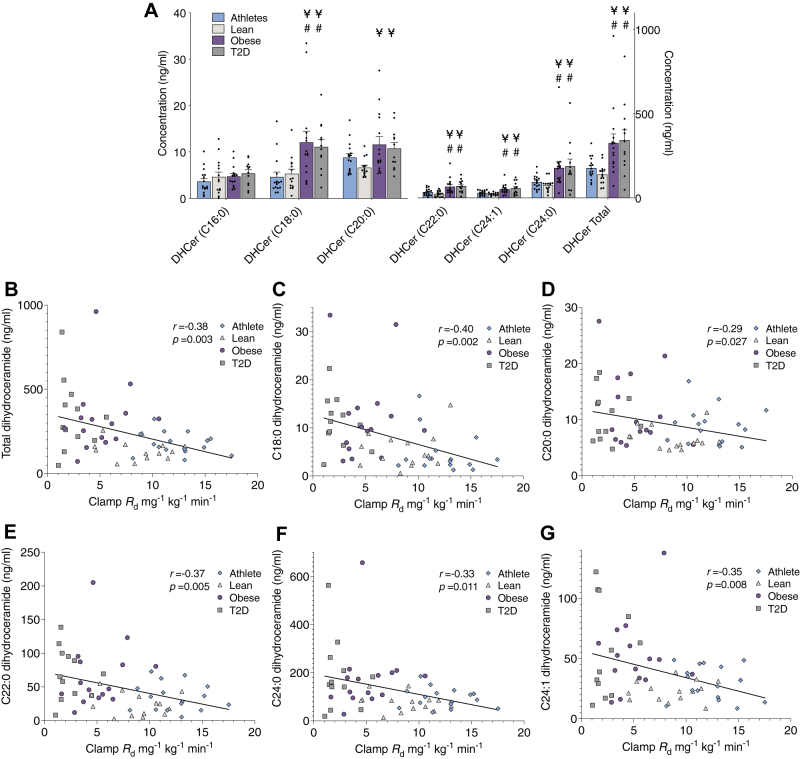
Serum dihydroceramides and relationships to insulin sensitivity. Serum dihydroceramide content (A) and relationship of total dihydroceramide content to insulin sensitivity (B) in endurance trained athletes, lean sedentary controls, and individuals with obesity without and with T2D. Relationships of individual dihydroceramide species to insulin sensitivity are shown for C18:0 (C), C20:0 (D), C22:0 (E), C24:0 (F), and C24:1 (G). Values are means ± SEM. ¥ = significantly different than lean *P* < 0.05, # = significantly different than athletes *P* < 0.05.

Total serum dihydroceramides were significantly greater in Ob and T2D compared with Lean and Ath (*P* = 0.0004; Fig. 3A). Total serum dihydroceramide was also significantly related to insulin sensitivity (Fig. 3B). This relationship was also significant for individual dihydroceramide species including C18:0, C20:0, C22:0, C24:0, and C24:1, as shown in Fig. 3C–G.

**Figure 4:**
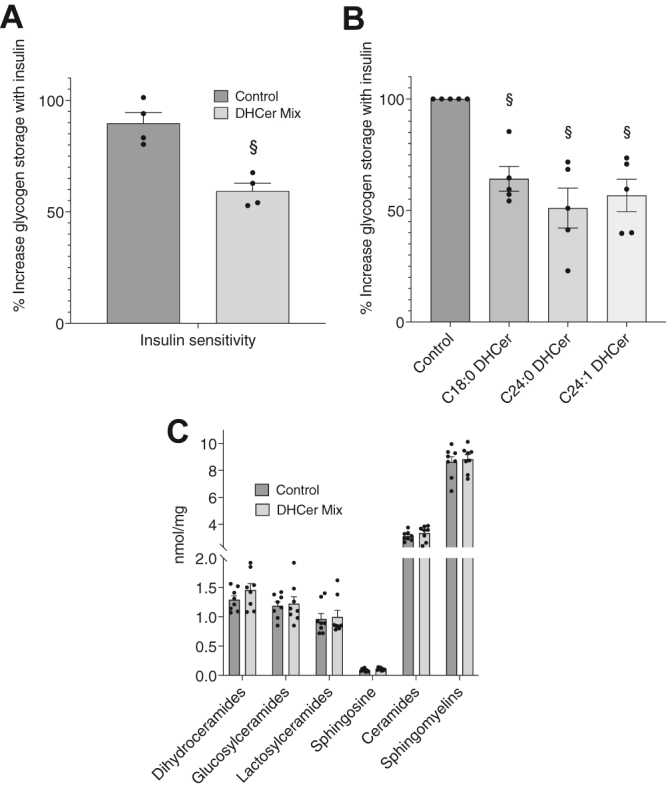
Effect of dihydroceramide-containing liposome administration on insulin sensitivity and cellular sphingolipid accumulation in human primary myotubes in vitro. Cells were treated for 3.5 h with POPC liposomes containing vehicle control (control) or a mixture of C18:0, C24:0, and C24:1 dihydroceramides (DHCer mix) (A) or individual dihydroceramides at the same concentration as the DHCer mix treatment (B). Effect of dihydroceramide administration on cellular sphingolipid content (C). Values are means ± SEM. § = significantly different than control, *P* < 0.05.

To evaluate if dihydroceramides could cause insulin resistance in vitro, we performed an insulin-stimulated glycogen synthesis assay in human primary myotubes treated with a mixture of dihydroceramides at a similar concentration observed in the serum of individuals with obesity, with and without T2D. We selected three of the dihydroceramide species that showed significant higher serum levels in individuals with obesity and T2D and used concentrations that reflected the average between the two groups in our in vitro experiments. We prepared phospholipid liposome vesicles containing the dihydroceramide mix to mimic in vivo dihydroceramide delivery to muscle and avoid precipitation in the extracellular hydrophilic environment. When primary skeletal muscle cells were treated with POPC liposomes containing a mixture of C18:0, C24:0, and C24:1 dihydroceramides, we observed a significant decrease in insulin-stimulated glycogen synthesis (34 ± 2%; *P* = 0.0005) compared with cells pretreated with POPC-only control liposomes (Fig. 4A). When we administered C18:0, C24:0, and C24:1 dihydroceramide liposomes individually, we observed a significant reduction in glycogen synthesis, compared with control, for all the individual species tested (C18:0, 35.8 ± 5.5%, *P* = 0.003; C24:0, 49 ± 9%, *P* = 0.005; C24:1, 43.3 ± 7.3%, *P* = 0.004) (Fig. 4B).

To evaluate the potential effect of extracellular dihydroceramide administration on intracellular sphingolipid levels, we measured ceramide, dihydroceramide, glucosylceramide, lactosylceramide, sphingosine, and sphingomyelin concentrations in untreated skeletal muscle cells or cells treated with control or dihydroceramide liposomes. As shown in Fig. 4C, extracellular delivery of dihydroceramide liposomes did not cause significant changes in intracellular level of these sphingolipids. These results suggest that, under these in vitro experimental conditions, the observed effects on cell insulin sensitivity are caused by extracellular dihydroceramides and are not affected by changes in other intracellular sphingolipids, such as ceramides, that are known to affect insulin resistance and would therefore make these data hard to interpret.

**Figure 5:**
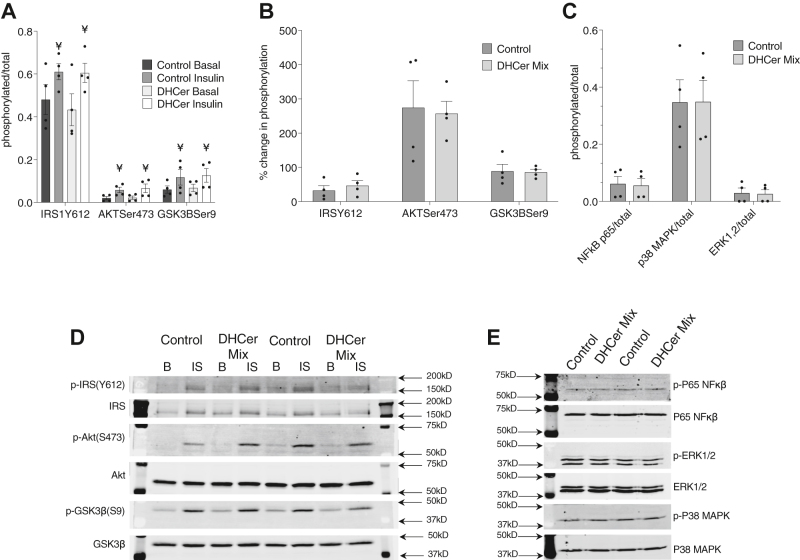
Effect of dihydroceramide administration on basal and insulin-stimulated signaling and inflammation in cultured primary human myotubes. Basal versus insulin-stimulated insulin signaling (A), relative changes in phosphorylation of downstream effectors of insulin signaling (B), inflammatory response (C), and representative blots for insulin signaling (D), and inflammatory response (E) in human skeletal muscle cells treated with POPC control liposomes (control), or with POPC liposomes containing a mixture of C18:0, C24:0, and C24:1 dihydroceramides (DHCer mix). Values are means ± SEM. ¥ = significantly different than basal, *P* < 0.05.

Treatment of human primary myotubes with a mixture of dihydroceramide liposomes, at the same experimental conditions that caused a significant decrease in insulin-stimulated glycogen accumulation, did not result in any significant changes in key nodes of insulin-stimulated insulin signaling including phosphorylation of IRS1(Tyr612), AKT(Ser473), and GSK-3β(Ser9) in response to insulin (Fig. 5A, B). We also wanted to measure common inflammatory response pathways that are known to attenuate insulin sensitivity in skeletal muscle in order to evaluate changes in cell signaling that could explain the insulin resistance observed. However, we did not observe significant differences in phosphorylation of proinflammatory transcription factor NF-κB p65, p38 MAPK, and p44/42 MAPK (ERK1/2) (Fig. 5C). Representative gels for insulin signaling and inflammatory response in vitro are shown in Fig. 5D, E. The full gels from which the images of Fig. 5D, E were spliced for presentation purposes can be found in supplemental Figs. S6 and S7, respectively. These results suggest that, in these experimental conditions, the effects on insulin sensitivity observed in myotubes after extracellular DHCer administration are not caused by alterations in insulin signaling or an inflammatory response in vitro.

## Discussion

The main findings from this study are that plasma dihydroceramide content is significantly greater in individuals with obesity with and without T2D, plasma ceramide and dihydroceramide content are negatively related to insulin sensitivity, and that dihydroceramide administration to myotubes decreased insulin sensitivity in vitro, consistent with a causal role in promoting whole-body insulin resistance. These data agree with the growing body of literature showing that plasma sphingolipids are important factors influencing the risk of insulin resistance and CVD. Our results suggest that interventions designed to decrease plasma dihydroceramide content may help combat obesity-induced insulin resistance.

Our results agree with data in the literature showing a relationship between serum ceramide concentration and insulin resistance in humans (1, 2, 5, 13, 15, 23). Plasma ceramide content decreases after bariatric surgery, the extent of which predicts the increase in insulin sensitivity (13). There is a growing consensus that specific serum ceramide species may play more deleterious roles in insulin resistance, specifically C16:0 and C18:0 species (2, 5, 12). Our data support this concept of the importance of species as we found that C18:0, C20:0, and C22:0 serum ceramides were inversely related to insulin sensitivity with the strongest relationship seen for C18:0 ceramide. These data corroborate findings from previous findings from our laboratory and others (2, 5). Explanations for why specific ceramide species are more negative than others are not known. However, it is possible that specific plasma ceramide species impact insulin sensitivity via incorporation into muscle ceramide species (15), where specific species are thought to be more potent in inducing insulin resistance (29, 30, 31, 32, 33).

Previous studies found that C16:0, C18:0, and C20:0 serum ceramide as an absolute concentration or relative to C24:0 significantly predicted CVD, which in some cases was a better predictor than LDL-cholesterol (6, 9, 12). For example, the C18:0 ceramide content in plasma was associated with a 31% increase in major adverse cardiovascular events in the FinRisk study (6). Inclusion of C18:0 ceramide provided increased predictive power for cardiovascular risk above that for LDL or HDL only. Mechanisms responsible for increased CVD risk with C18:0 ceramide are not known. However, the observation that atherosclerotic lesions that contain LDL are enriched in ceramide relative to plasma LDL and promote LDL aggregation (16) may provide some insight.

There are other serum sphingolipids that have been associated with insulin resistance in humans including sphinganines, glucosylceramides, lactosylceramides, gangliosides, and sphingomyelins. Administration of sphinganine, the precursor for dihydroceramide synthesis, can decrease skeletal muscle insulin sensitivity (34). Decreased glucosylceramide synthesis has been linked to decreased atherosclerosis in mice (35) and increased insulin sensitivity in vitro (36). Lactosylceramide species have also been linked to increased CVD risk (18), and decreased ganglioside formation has also been associated with increased insulin sensitivity and reduced risk factors for CVD (37, 38). Previous studies also showed that plasma sphingomyelin was related to CVD risk as well as subclinical CVD markers in humans (7, 39). Nevertheless, our data show no difference in plasma sphinganine, hexosylceramide, lactosylceramide, ganglioside, and sphingomyelin content across the spectrum of insulin sensitivity in humans. Taking our data together with previous publications, it is possible that, at least in humans, these sphingolipids are not likely to impact CVD risk through alterations in insulin sensitivity but rather may impact the composition of ceramides deposited in atherosclerotic lesions (16, 18) or influence plaque vulnerability (9).

The main observation from this study is the strong relationship between serum dihydroceramide content and insulin sensitivity in humans spanning the range of metabolic health. These data agree with the literature, which shows that total plasma dihydroceramide correlates with waist circumference (22) and was the strongest predictor in the plasma lipidome for the development of T2D 5 years before diagnosis (21). Total plasma dihydroceramide concentration was most different between individuals at time of diagnosis of T2D (21), as well as in cross-sectional studies comparing individuals with and without T2D (20), and nonhuman primates with prediabetes and T2D compared with controls (40). Therefore, our results recapitulate the strong relationship between circulating dihydroceramides and decreased insulin sensitivity. Beyond alterations in insulin sensitivity, dihydroceramides accumulate in atherosclerotic plaques and relate to plaque instability (41), and serum dihydroceramides are also linked to increased CVD risk (42). Thus, the literature supports that plasma dihydroceramide may be a marker of metabolic disease, CVD, and insulin resistance, but no studies have shown that plasma dihydroceramides cause insulin resistance.

We developed an in vitro protocol to deliver dihydroceramides to differentiated myotubes. Using phospholipid liposomes to mimic delivery in vivo, we administered three of the most abundant dihydroceramide species detected in serum to primary myotubes. Our in vitro data provide the first direct evidence that dihydroceramide administration to cells decreases insulin sensitivity. These results are not simply correlated, as our in vitro data show that administration of a mixture of dihydroceramides using liposomes caused a significant decrease in insulin sensitivity in human myotubes. The C18:0, C24:0, and C24:1 dihydroceramide species decreased insulin sensitivity to a similar extent, suggesting that the three most abundant plasma dihydroceramide species share similar potency with respect to promoting insulin resistance. Furthermore, in these experimental conditions, we could not observe any significant changes in intracellular level of ceramides, dihydroceramides, glucosylceramides, lactosylceramides, sphingosines, or sphingomyelins suggesting that the effects on cell insulin sensitivity are not because of dihydroceramide intracellular accumulation or conversion to ceramides. Through the activity of ceramidases, ceramides can be converted to sphingosines that can also promote insulin resistance (34). However, dihydroceramide administration to myotubes did not change sphingosine concentration, suggesting that alterations in ceramidase activity cannot explain the insulin resistance observed. Therefore, serum dihydroceramides appear to promote myotube insulin resistance. These data are consistent with the literature as well as with a growing amount of evidence showing that dihydroceramides are not an inert precursor to ceramide but rather potent signaling molecules that are related to development of prediabetes and T2D (20, 21, 23, 40).

Mechanisms explaining how plasma dihydroceramides decrease insulin sensitivity are currently unclear. Our data show that insulin signaling and inflammatory response are not altered when cells are treated with dihydroceramides at concentrations that cause a decrease in myotube insulin sensitivity in vitro. Our lipidomic data on myotubes exposed to extracellular dihydroceramides suggest that the effects on myotube insulin sensitivity are because of neither dihydroceramide uptake and conversion into ceramides via dihydroceramide desaturase (DES1) nor accumulation of dihydroceramide itself in muscle cells. Therefore, our data are consistent with a mechanism by which plasma dihydroceramides impact muscle cell insulin sensitivity via a receptor-based interaction.

Combined, our data show that recapitulation of physiological dihydroceramide delivery to cells can induce insulin resistance in myotubes in vitro and help explain the consistent findings in the literature that circulating dihydroceramides can predict the development of T2D. Thus, our data suggest a signaling role for serum dihydroceramides in inducing insulin resistance.

While the current article is focused on serum sphingolipids and insulin resistance, they are certainly not the only circulating lipid signals that can impact insulin sensitivity. Many other circulating lipids that have been implicated in promoting insulin resistance have not been measured in the current study. These include, but are not limited to, serum diacylglycerols, acylcarnitines, free fatty acids, eicosanoids, and oxidized phospholipids (43, 44, 45, 46, 47, 48).

To summarize, results from this study suggest that serum dihydroceramide concentration is significantly greater in individuals with obesity with and without T2D compared with lean individuals. Serum ceramide and dihydroceramide contents are negatively related to insulin sensitivity, and dihydroceramide administration to myotubes mimicking physiological serum delivery decreases insulin sensitivity in vitro. These data are consistent with a causal role of circulating dihydroceramides in promoting whole-body insulin resistance and help explain the large body of data suggesting that circulating dihydroceramide content can predict the development of diabetes. Therefore, these data suggest that interventions that decrease circulating dihydroceramide content may improve insulin sensitivity and decrease the risk of developing T2D.

## Data availability

The data described in this article will be shared upon request. Inquiries should be directed to and will be fulfilled by the lead contact Bryan C. Bergman (bryan.bergman@cuanschutz.edu), University of Colorado, Anschutz Medical Campus.

## Supplemental data

This article contains supplemental data.

## Conflict of interest

The authors declare that they have no conflicts of interest with the contents of this article.
