# Supplementary material for: Serum dihydroceramides correlate with insulin sensitivity in humans and decrease insulin sensitivity in vitro
Source: J Lipid Res. 2022 Aug 27;63(10):100270. doi: 10.1016/j.jlr.2022.100270 (PMC9508341; doi:10.1016/j.jlr.2022.100270)
Supplement: Supplemental Information [file mmc1.pdf]

SUPPLEMENTAL INFORMATION

Serum sphingolipids across the spectrum of insulin sensitivity in humans – importance of dihydroceramides

Simona Zarini[1], Joseph T. Brozinick[2], Karin A. Zemski Berry[1], Amanda Garfield[1], Leigh Perreault[1], Anna Kerege[1], Hai Hoang Bui[2], Phil Sanders[2], Parker Siddall[2], Ming Shang Kuo[2], Bryan C. Bergman[1]

[1]University of Colorado Anschutz Medical Campus, Aurora, CO

[2] Eli Lilly and Company, Indianapolis, IN

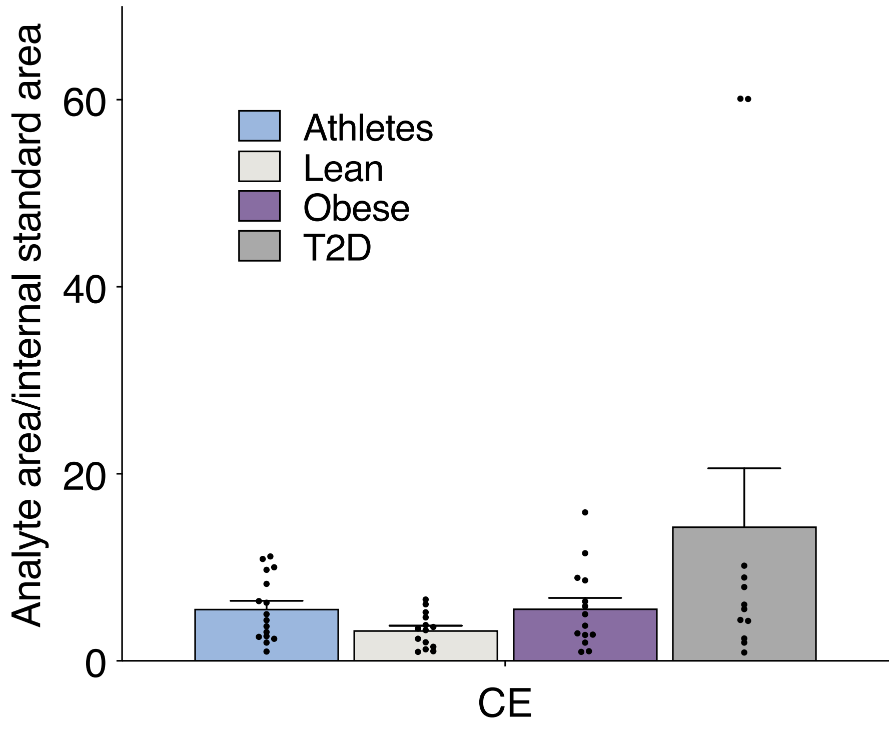

Supplemental Fig. S1. *Serum cholesterol esters (CE) in endurance trained athletes, lean sedentary controls, individuals with obesity without and with type 2 diabetes.* Values are means ± SEM and are expressed as ratio between the analyte and the internal standard areas in 1 mL of serum. Values are means ± SEM.

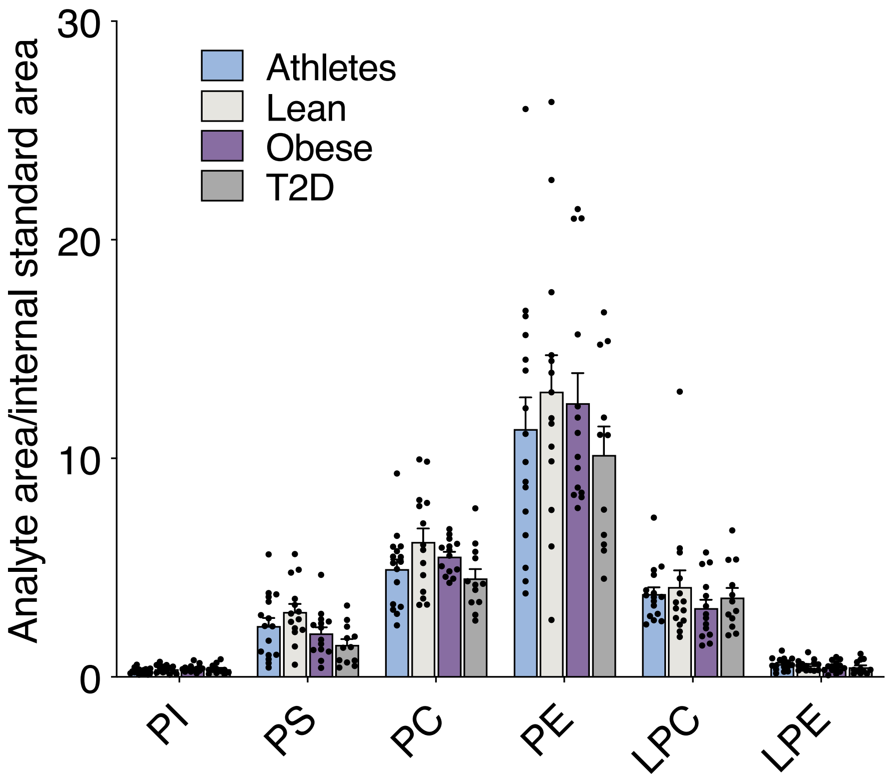

Supplemental Fig. S2. *Serum phospholipid and lysophospholipid content in endurance trained athletes, lean sedentary controls, individuals with obesity without and with type 2 diabetes.* Values are means ± SEM and are expressed as ratio between the analyte and the internal standard areas in 1 mL of serum. PI = phosphatidylinositol, PS = phosphatidylserine, PC = phosphatidylcholine, PE = phosphatidylethanolamine, LPC = lysophosphatidylcholine, LPE, lysophosphatidylethanolamine. Values are means ± SEM.

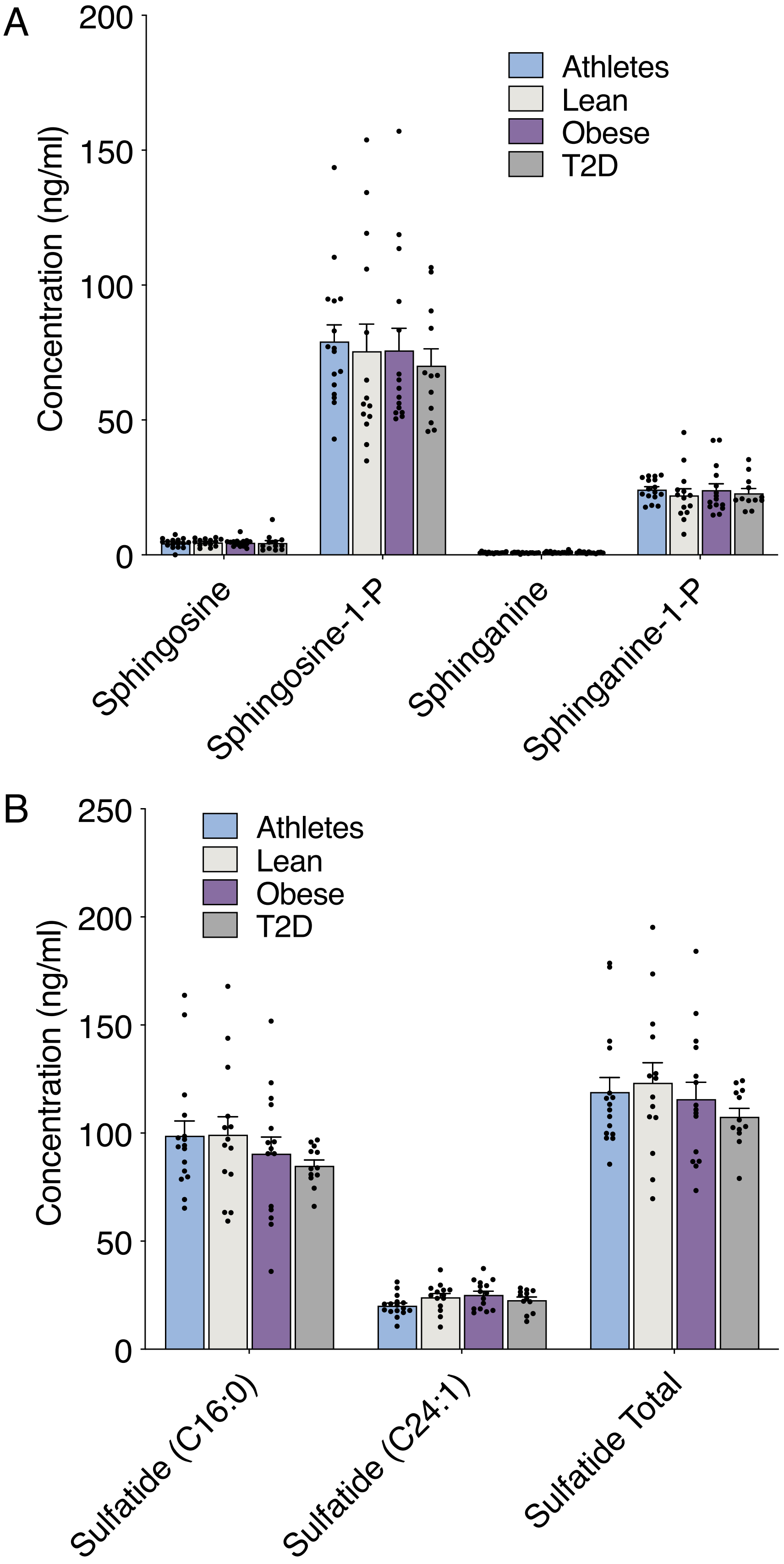

Supplemental Fig. S3. *Serum sphinganines, sphingosines (A), and sulfatides (B) in endurance trained athletes, lean sedentary controls, and individuals with obesity without and with type 2 diabetes.* Values are means ± SEM.

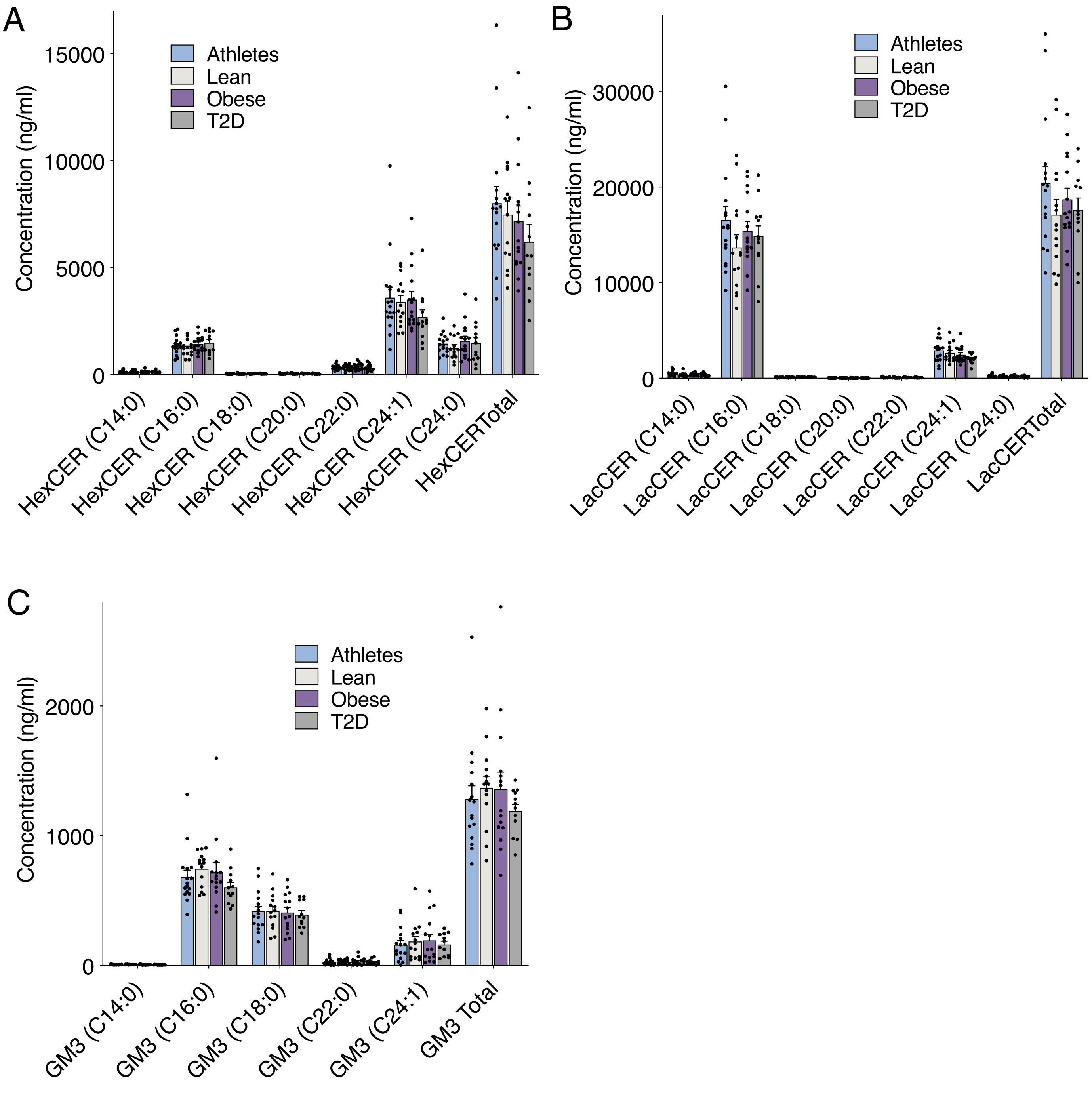

Supplemental Fig. S4. *Serum hexosylceramide (A) lactosylceramide (B), and ganglioside content (C) in endurance trained athletes, lean sedentary controls, individuals with obesity without and with type 2 diabetes.* Values are means ± SEM.

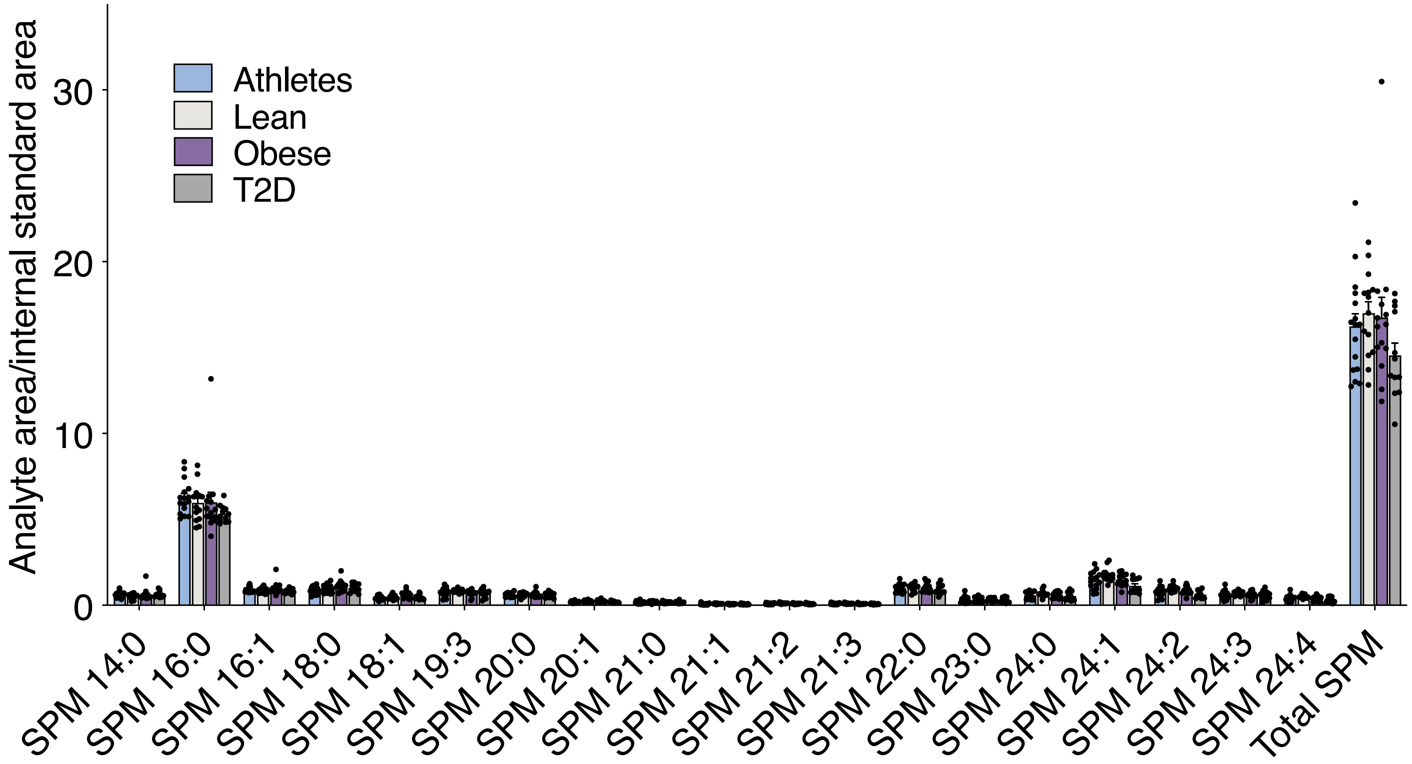

Supplemental Fig. S5. *Serum sphingomyelin content in endurance trained athletes, lean sedentary controls, and individuals with obesity without and with type 2 diabetes.* Values are means ± SEM and are expressed as ratio between the analyte and the internal standard areas in 1 ml of serum.

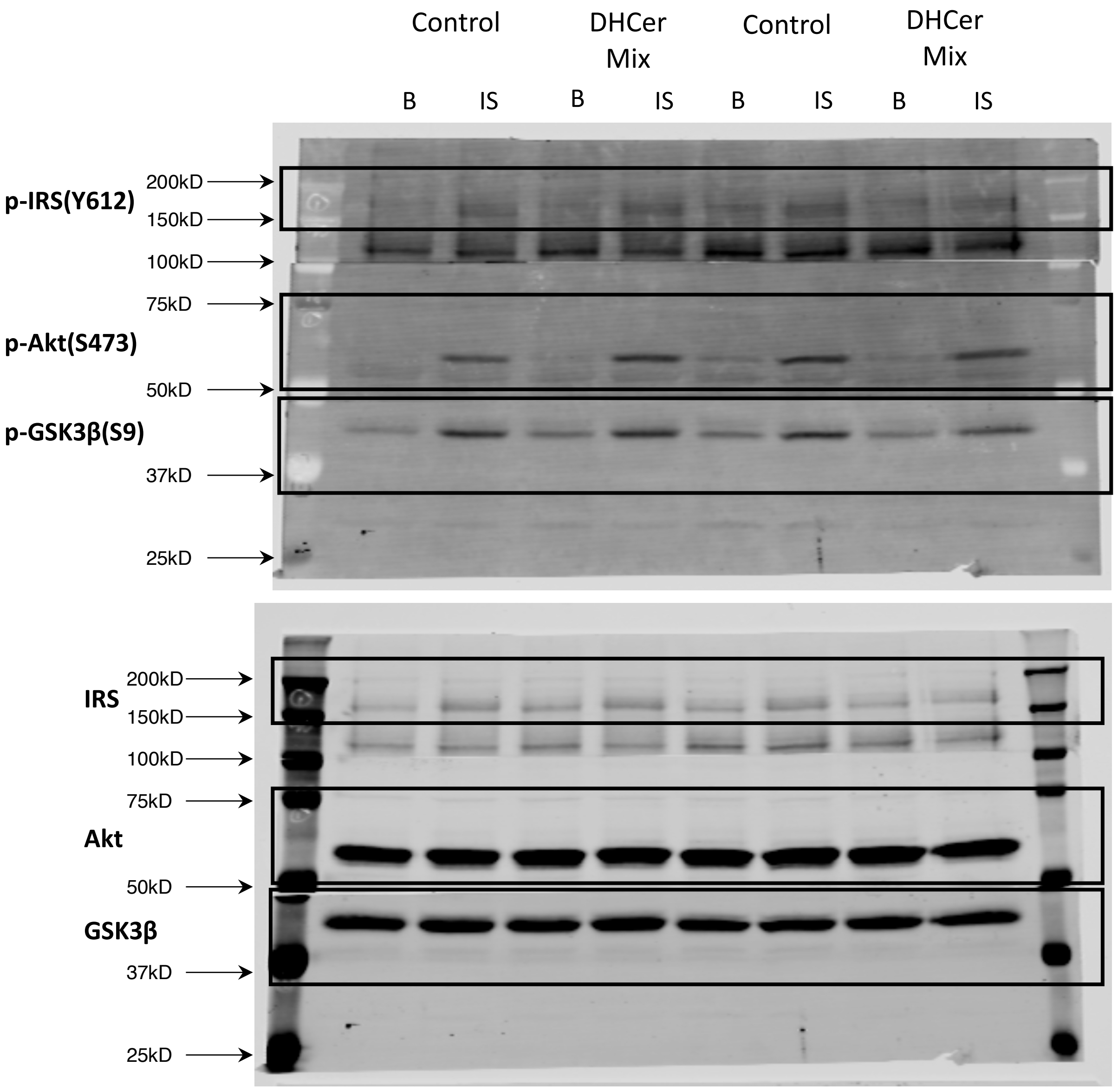

Supplemental Fig. S6. *Uncut gels from where cropped images for Figure 5D were obtained.*

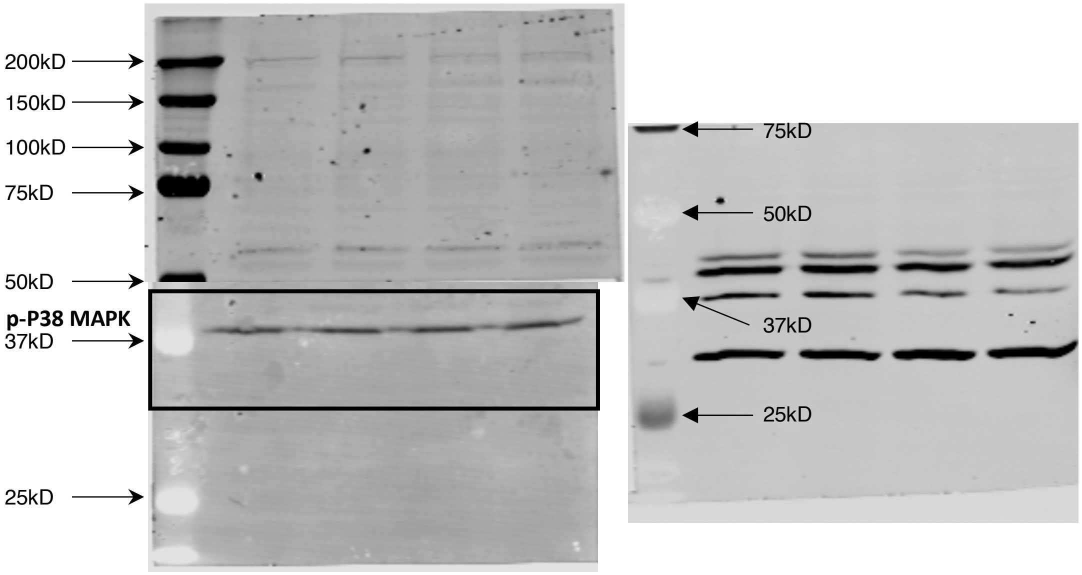
200kD
150kD
100kD
75kD
50kD
p-P38 MAPK
37kD
25kD
75kD
50kD
37kD
25kD

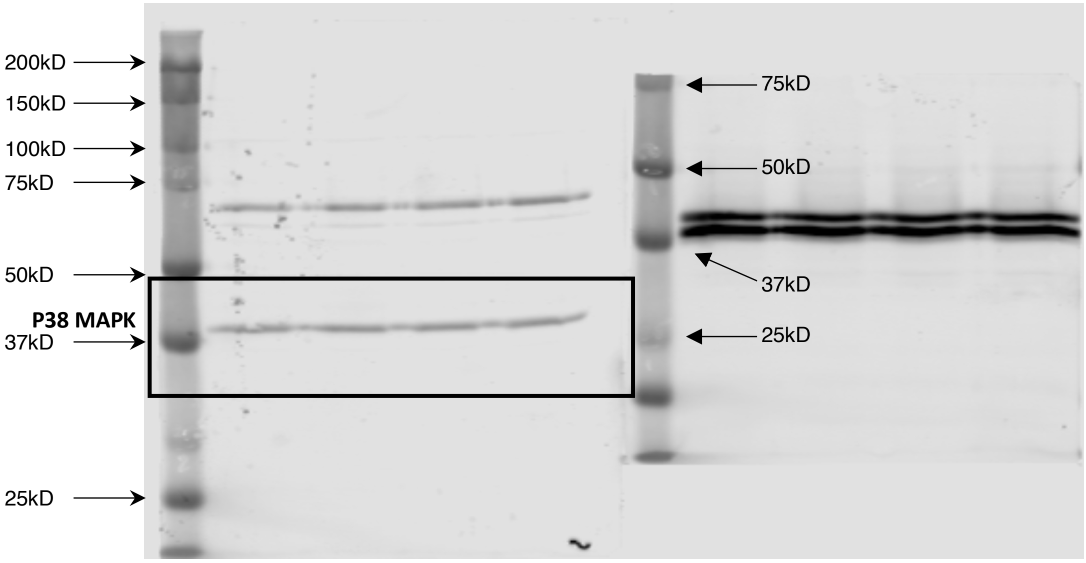
200kD
150kD
100kD
75kD
50kD
P38 MAPK
37kD
25kD
75kD
50kD
37kD
25kD

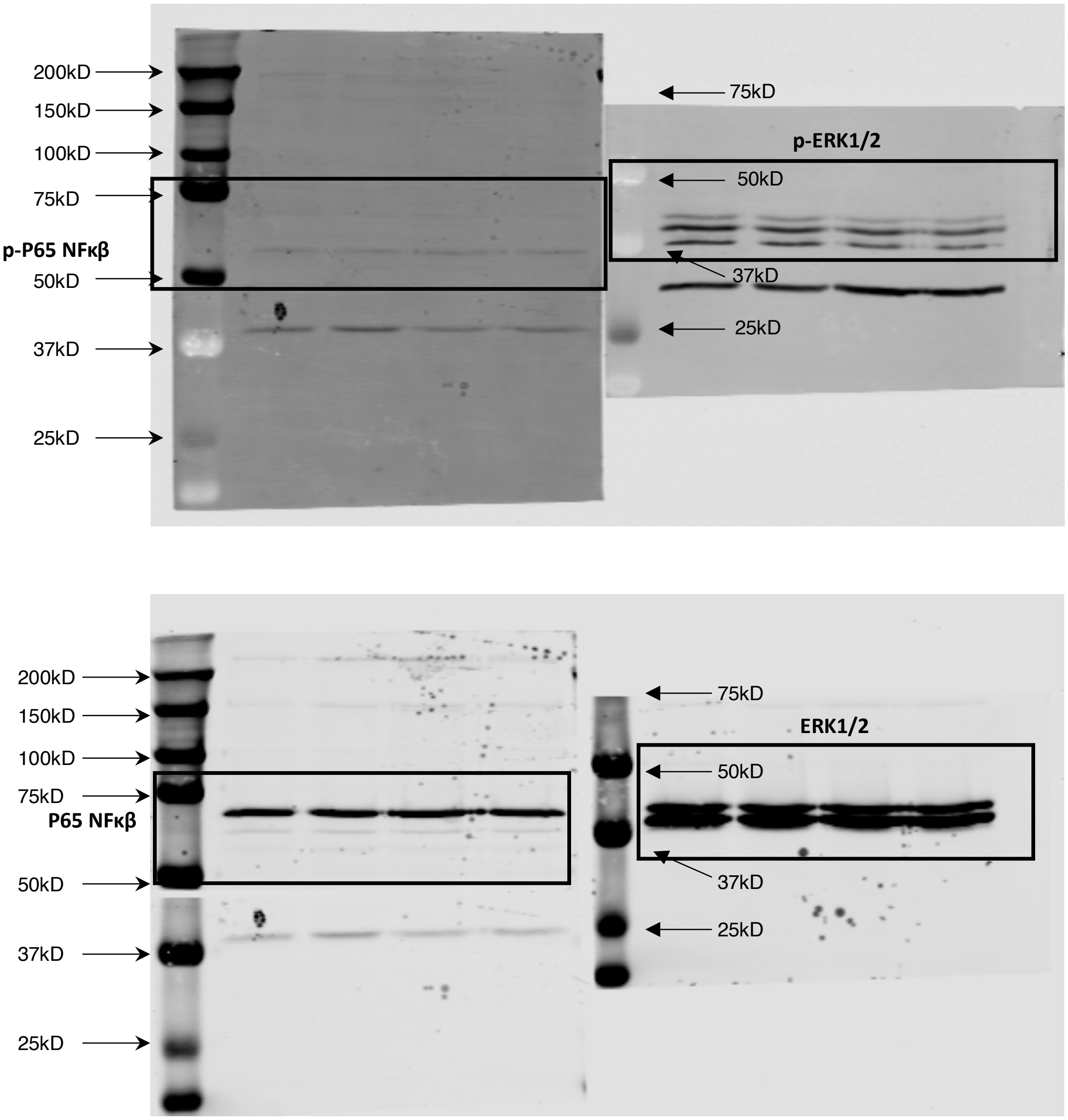

Supplemental Fig. S7. *Uncut gels from where cropped images for Figure 5E were obtained.*
